# The small heat shock protein HSPB1 protects mice from sepsis

**DOI:** 10.1038/s41598-018-30752-8

**Published:** 2018-08-21

**Authors:** Elise R. Breed, Carolyn A. Hilliard, Benyam Yoseph, Rohit Mittal, Zhe Liang, Ching-Wen Chen, Eileen M. Burd, Luke P. Brewster, Laura M. Hansen, Rudolph L. Gleason, Tej K. Pandita, Mandy L. Ford, Clayton R. Hunt, Craig M. Coopersmith

**Affiliations:** 1 Department of Surgery and Emory Critical Care Center, Emory University School of Medicine, Atlanta, GA Georgia; 2 Department of Radiation Oncology, Washington University School of Medicine, St. Louis, MO USA; 3 Department of Pathology and Laboratory Medicine, Emory University School of Medicine, Atlanta, GA Georgia; 4 Department of Surgery, Division of Vascular Surgery, Emory University School of Medicine, Atlanta, GA Georgia; 5 The Wallace H. Coulter Department of Biomedical Engineering, Georgia Institute of Technology, Atlanta, GA Georgia; 6 Department of Surgery and Emory Transplant Center, Emory University School of Medicine, Atlanta, GA Georgia; 7 Department of Radiation Oncology, The Houston Methodist Research Institute, Houston, TX USA

## Abstract

*In vitro* studies have implicated the small heat shock protein HSPB1 in a range of physiological functions. However, its *in vivo* relevance is unclear as the phenotype of unstressed HSPB1^−/−^ mice is unremarkable. To determine the impact of HSPB1 in injury, HSPB1^−/−^ and wild type (WT) mice were subjected to cecal ligation and puncture, a model of polymicrobial sepsis. Ten-day mortality was significantly higher in HSPB1^−/−^ mice following the onset of sepsis (65% vs. 35%). *Ex vivo* mechanical testing revealed that common carotid arteries from HSPB1^−/−^ mice were more compliant than those in WT mice over pressures of 50–120 mm Hg. Septic HSPB1^−/−^ mice also had increased peritoneal levels of IFN-γ and decreased systemic levels of IL-6 and KC. There were no differences in frequency of either splenic CD4^+^ or CD8^+^ T cells, nor were there differences in apoptosis in either cell type. However, splenic CD4^+^ T cells and CD8^+^ T cells from HSPB1^−/−^ mice produced significantly less TNF and IL-2 following *ex vivo* stimulation. Systemic and local bacterial burden was similar in HSPB1^−/−^ and WT mice. Thus while HSPB1^−/−^ mice are uncompromised under basal conditions, HSPB1 has a critical function *in vivo* in sepsis, potentially mediated through alterations in arterial compliance and the immune response.

## Introduction

The heat shock proteins (HSP) belong to a highly conserved family of molecular chaperones either demonstrated or postulated to have important roles in the host response to a number of pathophysiological stresses, including injury, oxidative damage, thermal stress, hypoxia, and infection^1–3^. A prominent and well characterized member of this family is HSPB1 (also known as mouse HSP25; human HSP27), which is present in a range of different tissues but at widely varied protein levels^4–6^. Levels of HSPB1 are also developmentally regulated during embryogenesis^7^. Despite high levels of HSPB1 in critical tissues, no obvious defects have been detected in any of three independent mouse HSPB1^−/−^ lines when housed under standard vivarium conditions^8–10^ even in aged mice^8,10^.

Notably, however, endogenous HSPB1 levels can be dramatically increased following stress or injury. Extensive studies in cultured cells have identified potential cellular mechanisms important to the immune response that could be regulated by HSPB1. In HeLa cells, HSPB1 interacts with IKKβ to inhibit tumor necrosis factor (TNF)-stimulated IκB degradation required for nuclear factor (NF)-κB dependent gene transcription^11^. Other investigators have found that HSPB1 does not inhibit interleukin (IL)-1-induced IκB degradation but does inhibit IKKβ^12^. In addition to NF-κB activation, HSPB1 has been shown to regulate cytokine mRNA stability. Knockdown of HSPB1 stabilizes TNF mRNA by disrupting AUF1 binding to AU-rich elements (ARE) sequences in the 3′ untranslated region^13^. Since similar elements are present in many cytokine or chemokine mRNAs, HSPB1 could impact levels for a wide range of immune factors. Notably, culturing Jurkat tumor cells and blood lymphocytes with a selective inhibitor of HSPB1 demonstrates a regulatory role of HSPB1 in the realization of apoptotic death as well as maintenance of functional activities of glutathione reductase and glutathione peroxidase^14^.

The significant role demonstrated for HSPB1 *in vitro* in cultured cells under stressed conditions coupled with the absence of a clear phenotype in HSPB1^−/−^ mice under non-stress housing conditions suggests that the major physiological contribution of HSPB1 may be in in stressed or injured tissues or hosts. Previously, we generated a new line of HSPB1^−/−^ mice and determined that HSPB1 loss is associated with decreased muscle fatigue resistance^10^. Here, we subjected HSPB1^−/−^ mice to cecal ligation and puncture (CLP), a commonly used model of polymicrobial sepsis^15,16^. Our results demonstrate that HSPB1 plays a critical role in protecting the host following sepsis, with markedly increased mortality in HSPB1^−/−^ mice following CLP.

## Materials and Methods

### Animals

**Figure 1:**
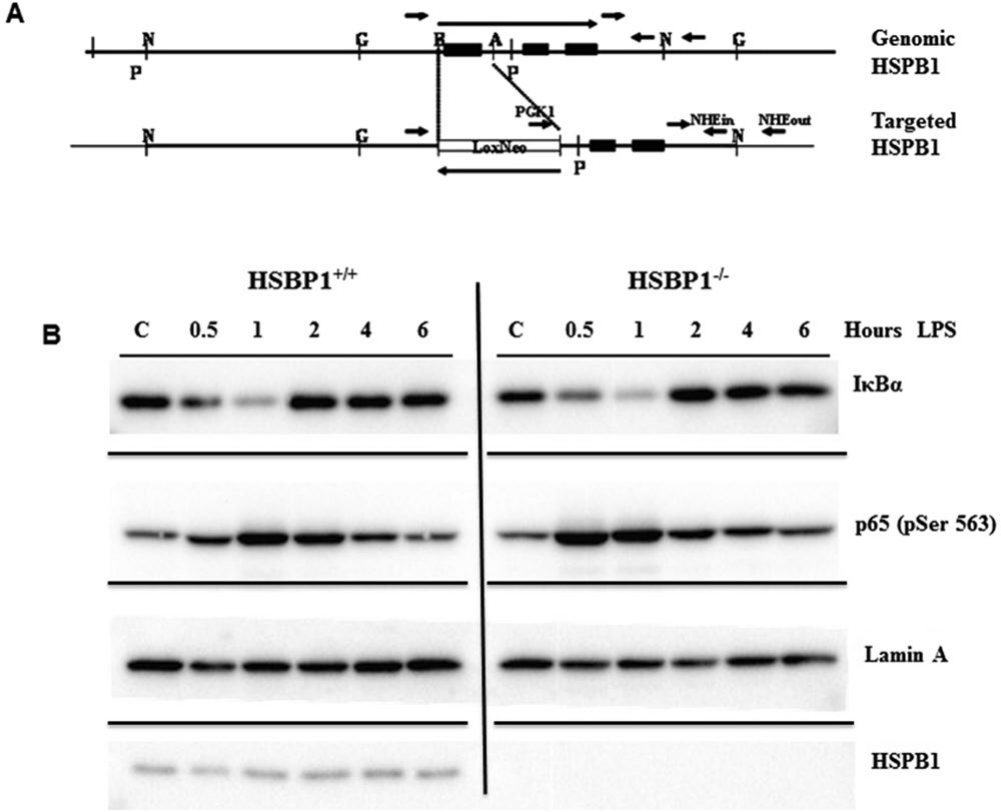
Effect of HSPB1 deletion on LPS-stimulated degradation of IκBα and p65 phosphorylation Mouse HSPB1 gene structure before and after replacement inactivation (**A**). Boxes represent HSPB1 exons, small arrows show oligonucleotide primers used to screen for correct gene replacement and large arrows show direction of transcription. Western blot analysis of cell extracts prepared from LPS stimulated HSPB1^+/+^ and HSPB1^−/−^ MEF cells demonstrates an HSPB1-independent near complete degradation of IκBα by 1 hour followed by recovery by 2 hours with a corresponding increase in RelA/p65 phosphorylation at Ser563 (**B**).

Six- to 18-week-old male and female mice in equal proportion were used for all experiments. Deletion of the HSPB1 gene was done by a targeted PGK-neo replacement as previously described^10^ and shown in Fig. 1. Homozygous HSPB1^−/−^ mice were maintained by in-crossing and are of mixed 129X1/SvJxC57Bl/6 background. Confirmation of genotype was performed by PCR analysis of isolated tail DNA. Embryonic fibroblast cell lines were prepared from day 13.5 embryos by standard protocols, genotyped and analyzed by Western blot analysis to confirm that the HSPB1^−/−^ genotype correlated with HSPB1 protein loss.

All knockout animals were compared to 129/SvJxC57BL/6 controls bred in the same facility. Animals were maintained on 12 hour light/dark cycles and had free access to food and water. Experiments were performed in accordance with National Institutes of Health guidelines for the Use of Laboratory Animals and were approved by the Washington University Institutional Animal Care and Use Committee (CRH was on faculty at Washington University when the mice were initially generated, protocol #20080146) and Emory University Institutional Animal Care and Use Committee (sepsis experiments were performed in CMC’s lab, protocol #DAR-200217).

### Western blot analysis

Western blot analysis was carried out on mouse embryonic fibroblast cell lines derived from HSPB1^+/+^ and HSPB1^−/−^ mice and grown in standard DMEM media supplemented with 10% fetal calf serum and 1X nonessential amino acids. Subconfluent cells were treated with 200 ng/ml lipopolysaccharide (LPS, *E*. *coli* O55:B5; SigmaAldrich) and cell extracts prepared from cells after 0.5 to 6 hours exposure. Twenty micrograms of protein was fractionated by SDS gel electrophoresis and transferred to PDVF membrane by standard techniques following by probing with the following primary antibodies: rabbit anti-IκB-α, Cell Signaling, Danvers, MA), rabbit anti-phospho-NF-κB p65 (Ser536) (Cell Signaling), mouse anti-Lamin A (Santa Cruz Biotechnology, Dallas, TX) and rabbit anti-HSP25 (Enzo Life Sciences, Farmingdale, NY). Secondary antibodies were horseradish perioxidase coupled goat anti-mouse or anti-rabbit (Jackson ImmunoResearch, West Grove, PA). Chemilumenscence (SuperSignal West Femto kit; Thermo Scientific, Norcross, GA) was captured by X-ray film.

### Sepsis model

Animals underwent cecal ligation and puncture, a model of polymicrobial, intra-abdominal sepsis^15,16^. Under isoflurane anesthesia, a midline abdominal incision was made. The cecum was then exteriorized, ligated 2 centimeters distal to the ileocecal valve, and punctured through-and-through with a 25-gauge needle. The cecum was returned to the abdominal cavity and the abdominal wall was closed in layers. All mice received fluid resuscitation and antibiotics to mimic the clinical management of sepsis^17^. Specifically, animals were given a subcutaneous injection of 1 ml of normal saline to compensate for fluid loss from insensible losses during laparotomy and vasodilation and vascular permeability from sepsis. In addition, all mice received ceftriaxone (25 mg/kg, Sigma-Aldrich, St. Louis, MO) and metronidazole (12.5 mg/kg, Apotex, Weston, FL) via intraperitoneal injection post-operatively and for 48 hours following surgery. Mice also received a single dose of buprenorphine (McKesson Medical, San Francisco, CA, USA). Animals were either sacrificed at 24 hours after CLP or followed 10 days for survival.

### Cylindrical biaxial testing of carotid arteries and composition of arteries

Bilateral common carotid arteries were harvested from mice and cylindrical biaxial biomechanical testing was performed as previously published^18,19^. Briefly, carotid arteries were harvested from mice at time of sacrifice, perfused with saline, isolated, excised, and preconditioned for testing *ex vivo*. Testing was performed in an incubator at 37 °C with 5% carbon dioxide in DMEM after the vessels were fully dilated using sodium nitroprusside. Pressure-diameter curves were generated and compliance curves were calculated as the change in diameter over change in pressure normalized to diameter. Testing included three loading and unloading pressurization cycles from 0 to 160 mmHg at constant axial stretch levels ranging from 1.3 to 1.9x. Force-length tests included three loading and unloading force-controlled cycles from 0 to 3 g at constant pressures across a blood pressure spectrum (0, 40, 60, 80, 100, and 120 mmHg). Elastin content was quantified as published^20^.

### Cytokine analysis

Whole blood was collected via cardiac puncture at time of sacrifice and centrifuged at 10,000 rpm for 10 minutes in serum separator tubes. Peritoneal fluid was obtained by lavaging the peritoneum with 3 ml sterile phosphate-buffered saline (PBS) and then centrifuged to obtain a supernatant. Both serum and peritoneal fluid cytokine concentrations were measured using a Bio-Plex Pro Mouse Cytokine Standard Group I Kit (Bio-Rad, Hercules, CA) per the manufacturer’s protocol. All samples were run in duplicate.

### Flow cytometric analysis

Spleens were removed from mice and single-cell suspensions were prepared by mashing the spleen using a 3-ml syringe plunger on a strainer (70 μM) and washing cells with PBS. Single cell suspensions were stained for flow cytometric analysis with anti-CD3-APC, anti-CD4-Pac Blue, anti-CD8-Pac Orange, Anti- B220-Per CP, anti-annexin-FITC, and anti PI-PE (BD Pharmingen, San Diego, CA). Blood and peritoneal fluid were also harvested and single-cell suspensions were prepared. Cells were stained with Gr-1-FITC, B220-Per CP, CD11b-APC, CD3-Pac Blue, CD11c-PE-Cy7; or CD4-Pac Blue, CD8-Pac Orange, Ly49D-FITC, NK1.1-PE, CD127-APC.

To measure production of cytokines on a per cell basis, splenocytes were stimulated with phorbol 12-myristate 13-acetate (PMA, 30 ng/mL) and ionomycin (400 ng/mL) in the presence of 10 μg/mL of Brefeldin A. After 18 hours, cells were surface stained with anti-CD4 and anti-CD8 and processed with an intracellular staining kit (BD Biosciences) according to manufacturer’s instructions. Intracellular antibodies included anti-interferon (IFN)-γ (eBioscience, San Diego, CA), anti-TNF, and anti-IL-2 (both BD Biosciences). Data were acquired on a LSR II multicolor flow cytometer (BD Biosciences) and analyzed using FlowJo software (TreeStar, San Carlos, CA).

### Bacterial cultures

Serum and peritoneal fluid were obtained as described above. Samples were serially diluted in sterile saline and plated on sheep blood agar plates. Plates were incubated overnight at 35 °C in 5% CO_2_ and examined for colony counts later.

### Intestinal epithelial apoptosis

Apoptotic cells in the intestinal epithelium were quantified in 100 contiguous and well-oriented crypt-villus units via active caspase-3 staining^21,22^. Intestinal sections were deparaffinized, rehydrated, and incubated in 3% hydrogen peroxide for 10 minutes. To facilitate antigen retrieval, slides were placed in antigen decloaker (Biocare Medical, Concord, CA) and heated in a pressure cooker for 45 minutes. Sections were blocked with 20% goat serum (Vector Laboratories, Burlingame, CA) and incubated with rabbit polyclonal anti–active caspase-3 (1:100; Cell Signaling Technology, Beverly, MA) overnight at 4 °C. Sections were then incubated with goat anti-rabbit biotinylated secondary antibody (1:200; Vector Laboratories) for 30 minutes at room temperature, followed by Vectastain Elite ABC reagent (Vector Laboratories) for 30 minutes at room temperature. Slides were developed with diaminobenzidine and counterstained with hematoxylin.

### Statistics

Data were analyzed using the statistical software program Prism 6.0 (Graphpad, San Diego, CA) and are presented as mean ± standard error of the mean or as box and whiskers plots with 25^th^ and 75^th^ percentile. All data were tested for Gaussian distribution using the D’Agostino-Pearson omnibus normality test. If data were found to have a Gaussian distribution, comparisons were performed using the Student t test. If data did not have a Gaussian distribution, comparisons were performed using the Mann–Whitney U test. Survival studies were analyzed using the log-rank test. A *p* value < 0.05 was considered statistically significant.

## Results

### Loss of HSPB1 does not alter LPS induced IκB degradation or p65 phosphorylation

Activation and signaling through the NF-κB pathway is an important factor in cell survival under adverse or injury conditions. *In vitro* studies have suggested that several steps in the pathway are subject to HSPB1 regulation. To examine the possible relationships between the NF-κB pathway, HSPB1 and *in vivo* injury responses, the HSPB1 gene was inactivated in mice by standard gene targeting procedures (Fig. 1A). Embryonic fibroblast cell lines (MEF) were established from WT (HSPB1^+/+^) and HSPB1^−/−^ embryos. Stimulation of either HSPB1^+/+^ or HSPB1^−/−^ MEF cells with LPS resulted in nearly complete degradation of IκBα by 1 hour followed by recovery due to re-synthesis by 2 hours (Fig. 1B). A corresponding increase in RelA/p65 phosphorylation at Ser563 was observed. The activity of the NF-κB transcription complex is increased by p65 subunit ser563 phosphorylation and this is consistent with IκBα recovery which is dependent upon NF-κB mediated transcription. A similar pattern of IκBα degradation followed by re-synthesis was seen in TNF (10 ng/ml) or IL-1β (1 ng/ml) stimulated MEF cells (data not shown) indicating that lack of HSPB1 does not alter IκBα degradation or NF-κB activation in general.

### Loss of HSPB1 increases mortality from sepsis

**Figure 2:**
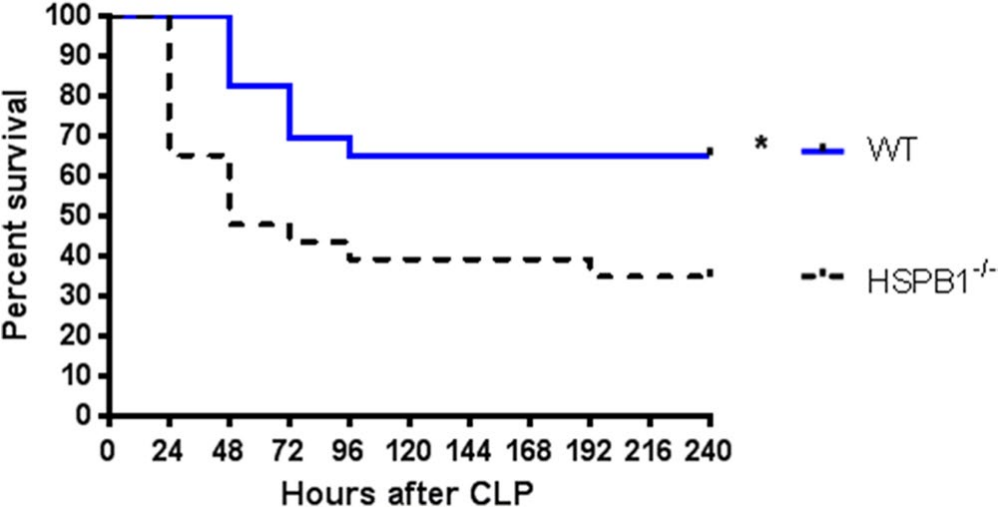
Effect of HSBP1 deletion on survival. Septic HSPB1^−/−^ mice had significantly higher 10-day mortality than septic WT mice (p = 0.016, n = 23/group).

WT mice exhibited a 35% ten-day mortality following CLP. In contrast, HSPB1^−/−^ mice had a significant increase in mortality, with 65% of knockout mice dying after induction of sepsis (Fig. 2).

### Loss of HSPB1 increases carotid artery compliance following sepsis

**Figure 3:**
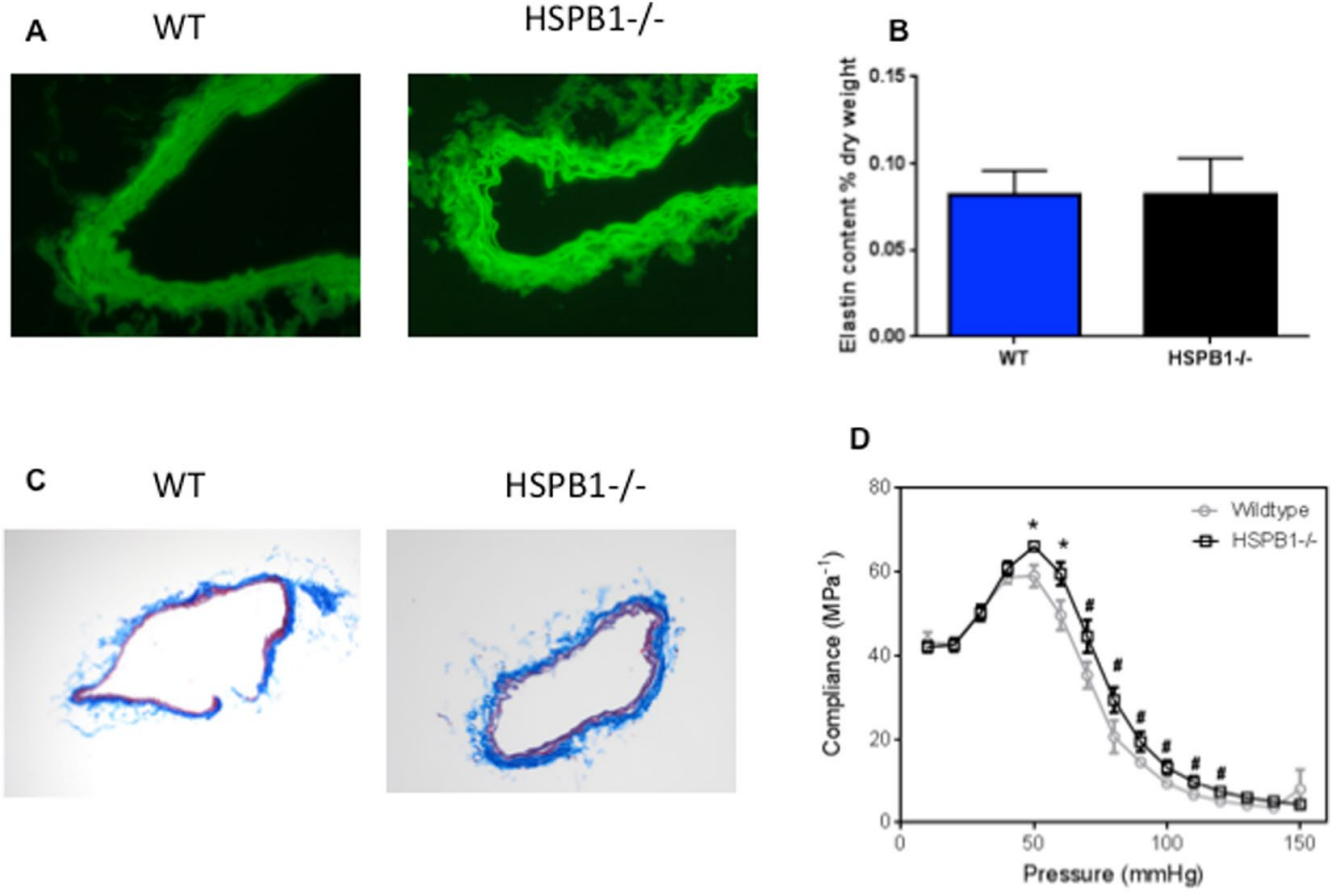
Effect of HSBP1 deletion on carotid artery histology and compliance. Autofluorescent analysis did not demonstrate differences in elastin architecture or content between septic HSPB1^−/−^ mice and WT mice (**A**,**B**, p = 0.90, n = 3/group). Similarly trichrome staining did not identify gross differences in collagen content between HSPB1^−/−^ and WT carotid arteries (**C**). In contrast carotid arteries from HSPB1^−/−^ mice were significantly more compliant than carotid arteries from WT mice from 50 mmHg to 120 mmHg (**D**).

Elastin organization and content in the carotid artery was similar between WT and HSPB1^−/−^ mice (Fig. 3A,B). In addition, arterial sections did not manifest aberrant extracellular matrix compositions as assessed by histology (Fig. 3C). However the compliance of carotid arteries in HSPB1^−/−^ mice was significantly increased over a broad range of blood pressures (50–120 millimeters of mercury (mm Hg) compared to carotid arteries from WT mice (Fig. 3D).

### Loss of HSPB1 alters both local and systemic cytokines following sepsis

**Figure 4:**
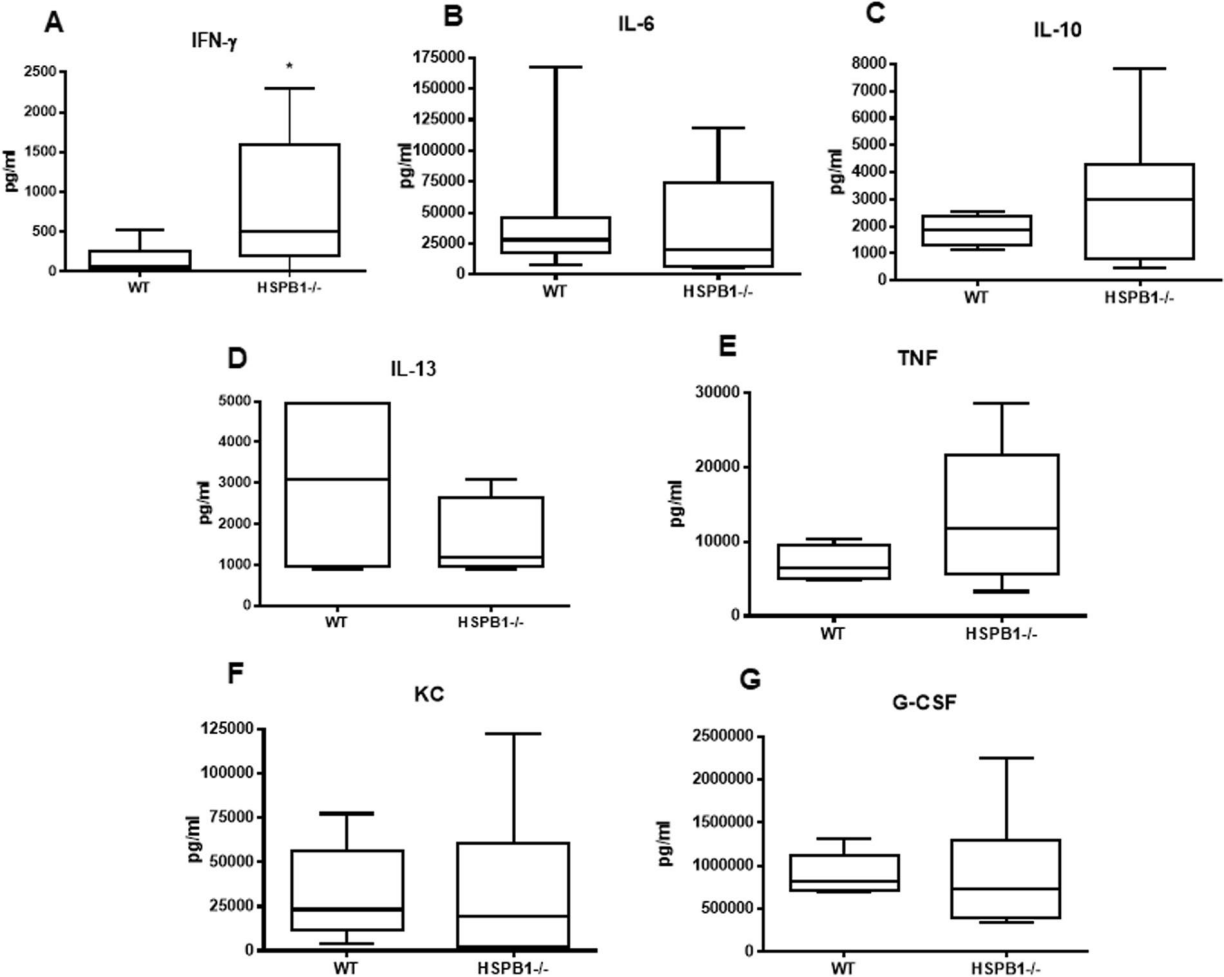
Effect of HSBP1 deletion on peritoneal fluid cytokines. IFN-γ was higher in septic HSPB1^−/−^ mice than WT mice (**A**, p = 0.034, n = 7–8/group). In contrast, there was no difference in IL-6 (**B**, p = 0.69, n = 7/group), IL-10 (**C**, p = 0.31, n = 4–7/group), IL-13 (**D**, p = 0.57, n = 4/group), TNF (**E**, p = 0.29, n = 4–5/group), KC (**F**, p = 0.68, n = 8–10/group) or G-CSF (**G**, p = 0.54, n = 8–11/group).

**Figure 5:**
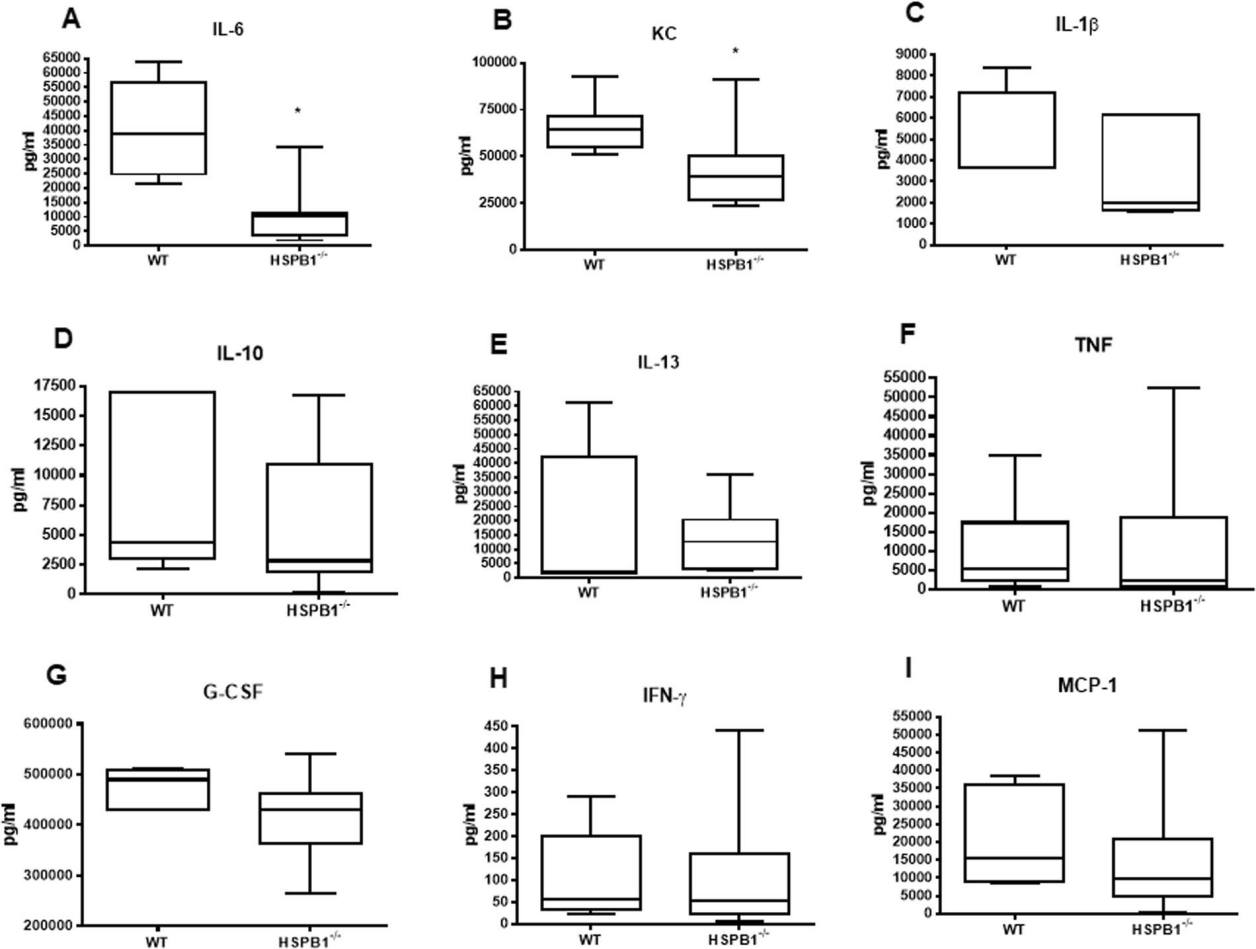
Effect of HSBP1 deletion on systemic cytokines. Serum IL-6 was lower in septic HSPB1^−/−^ mice than WT mice (**A**, p = 0.01. n = 5–7/group). KC was also lower in septic HSPB1^−/−^ mice (**B**, p = 0.01, n = 6–9/group). In contrast, there was no difference in IL-1β (**C**, p = 0.23, n = 4–6/group), IL-10 (**D**, p = 0.22, n = 6–11/group), IL-13 (**E**, p = 0.5, n = 5–7/group), TNF (F, p = 0.33, n = 6–8/group), G-CSF (**G**, p = 0.056, n = 7–12/group), IFN-γ (**H**, p = 0.83, n = 5–8/group) and MCP-1 (I, p = 0.28, n = 7–11/group).

Local peritoneal levels of IFN-γ were increased in HSPB1^−/−^ mice (Fig. 4A) while IL-6, IL-10, IL-13, TNF, KC and granulocyte colony-stimulating factor (G-CSF) levels were similar between knockout and WT mice (Fig. 4B–G). In contrast, systemic levels of IL-6 and KC levels were reduced in HSPB1^−/−^ mice (Fig. 5A,B) while there were no statistically significant differences in IL-1β, IL-10, IL-13, TNF, G-CSF, IFN-γ, and MCP-1 levels between WT and HSPB1^−/−^ mice (Fig. 5C–I).

### Loss of HSPB1 causes significant decreases in stimulated cytokine production from CD4^+^ and CD8^+^ splenocytes following sepsis

**Figure 6:**
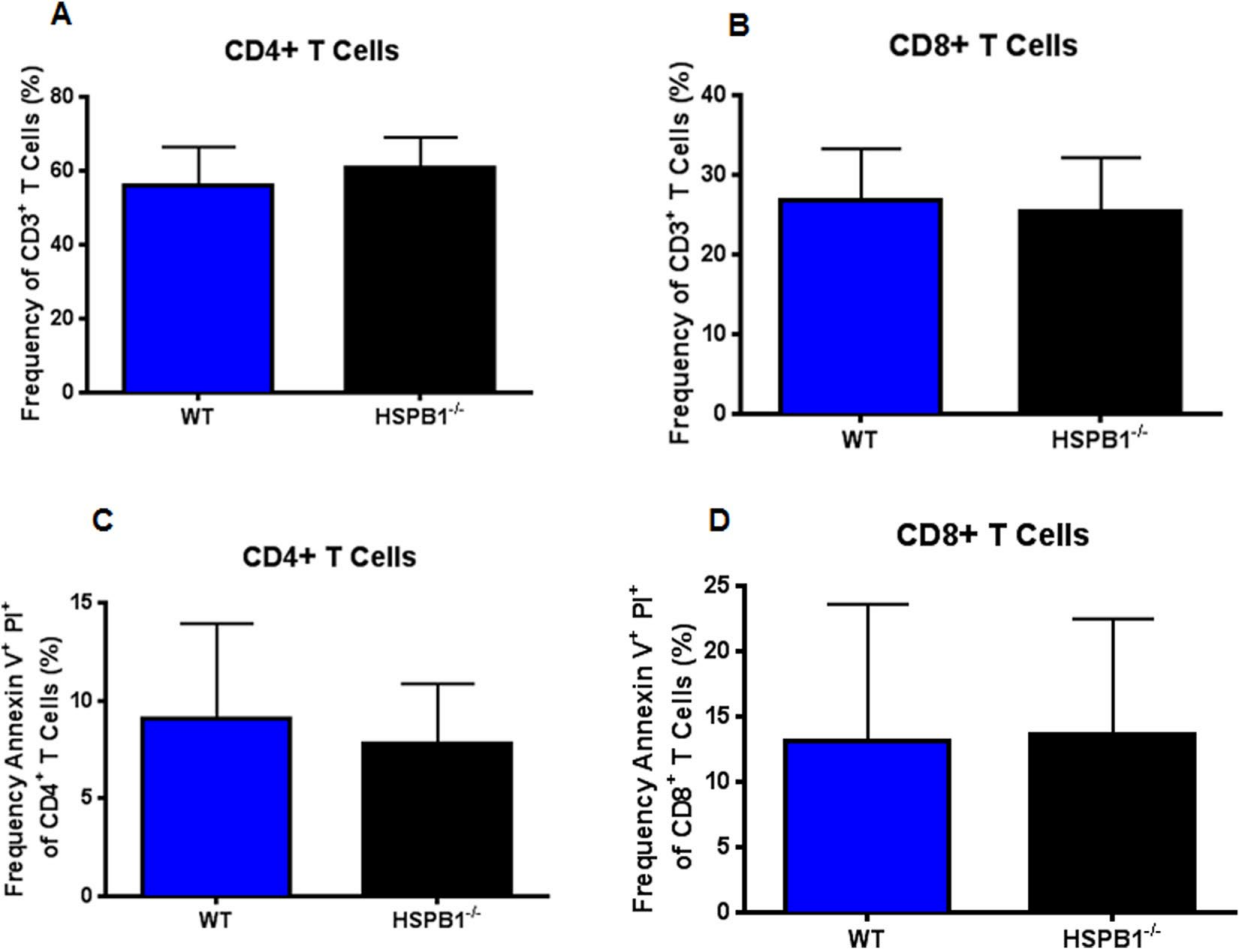
Effect of HSBP1 deletion on T cell frequency and apoptosis. Frequency of both CD4^+^ T cells (**A**, p = 0.28) and CD8^+^ T cells (**B**, 0.61) was similar between septic HSPB1^−/−^ and WT mice. Apoptosis was also similar for CD4^+^ T cells (**C**, p = 0.52) and CD8^+^ T cells (**D**, 0.84) between septic HSPB1^−/−^ and WT mice. (n = 10/group, all panels).

No differences were detected in frequency of splenic CD4^+^ T cells or CD8^+^ T cells between septic WT and HSPB1^−/−^ mice (Fig. 6A,B). Similarly, apoptotic cells were similar in CD4^+^ T cells and CD8^+^ T cells between WT and knockout mice (Fig. 6C,D).

**Figure 7:**
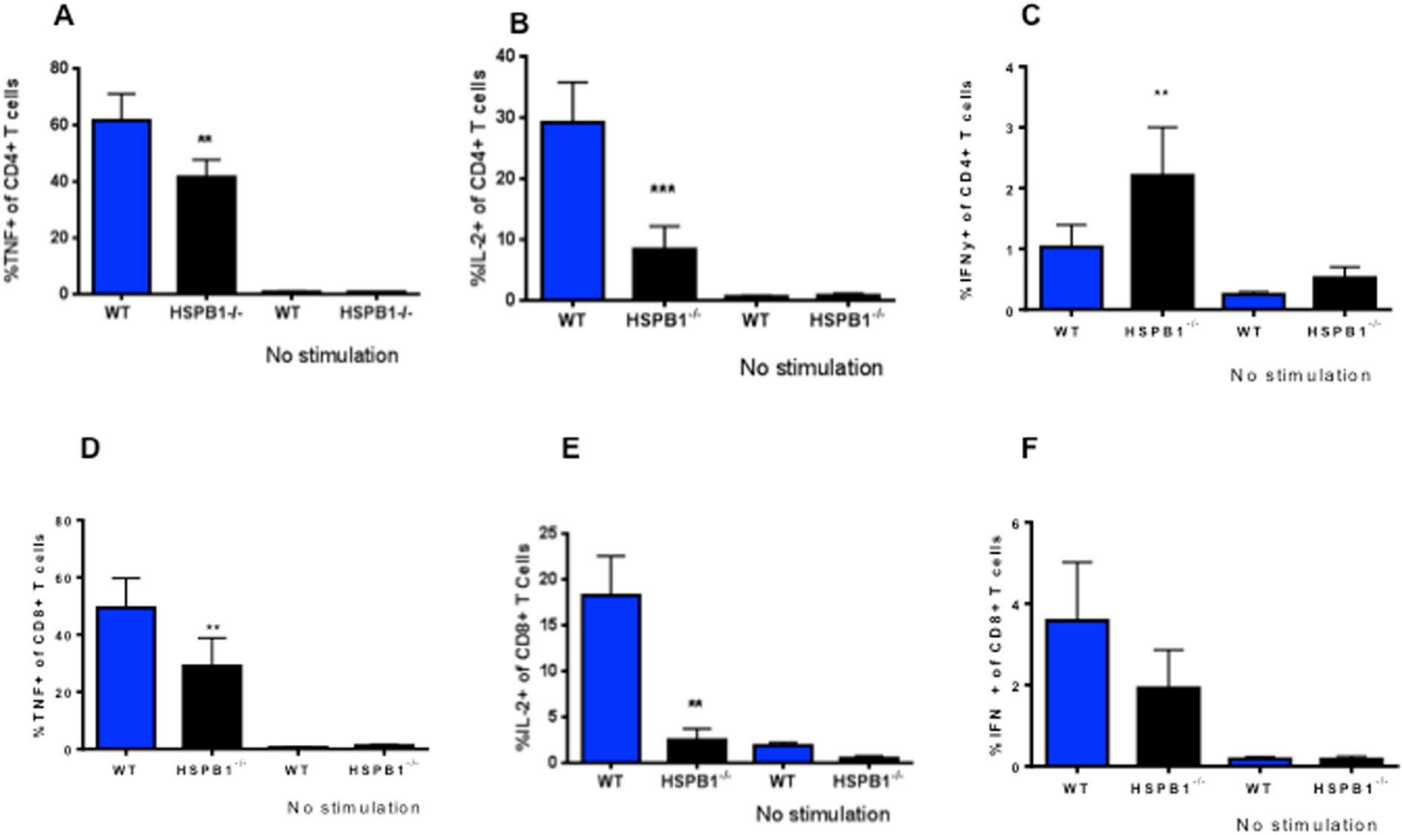
Effect of HSBP1 deletion on T cell stimulated cytokine production. TNF production (**A**, p = 0.009) and IL-2 production (**B**, p = 0.002) were decreased in splenic CD4^+^ T cells of HSPB1^−/−^ mice stimulated *ex vivo*. In contrast, IFN-γ production was elevated in CD4^+^ T cells of HSPB1^−/−^ mice (**C**, p = 0.009). TNF production (**D**, p = 0.004) and IL-2 production (**E**, p = 0.002) were also decreased in splenic CD8^+^ T cells of HSPB1^−/−^ mice stimulated *ex vivo* while IFN-γ production was unchanged (**F**, p = 0.13). N = 10/group, all panels).

Splenic CD4^+^ T cells isolated from septic HSPB1^−/−^ mice exhibited a marked decrease in TNF (Fig. 7A) and IL-2 production (Fig. 7B) as well as a modest increase in IFN-γ production ((Fig. 7C) following *ex vivo* stimulation as compared to those isolated from septic WT mice. Splenic CD8^+^ T cells isolated from septic HSPB1^−/−^ mice also exhibited a marked decrease in TNF and IL-2 production (Fig. 7D,E) following *ex vivo* stimulation as compared to those isolated from septic WT mice. In contrast, no significant changes in IFN-γ production (Fig. 7F) were detected.

### Loss of HSPB1 does not impact bacterial burden but decreases intestinal epithelial apoptosis following sepsis

**Figure 8:**
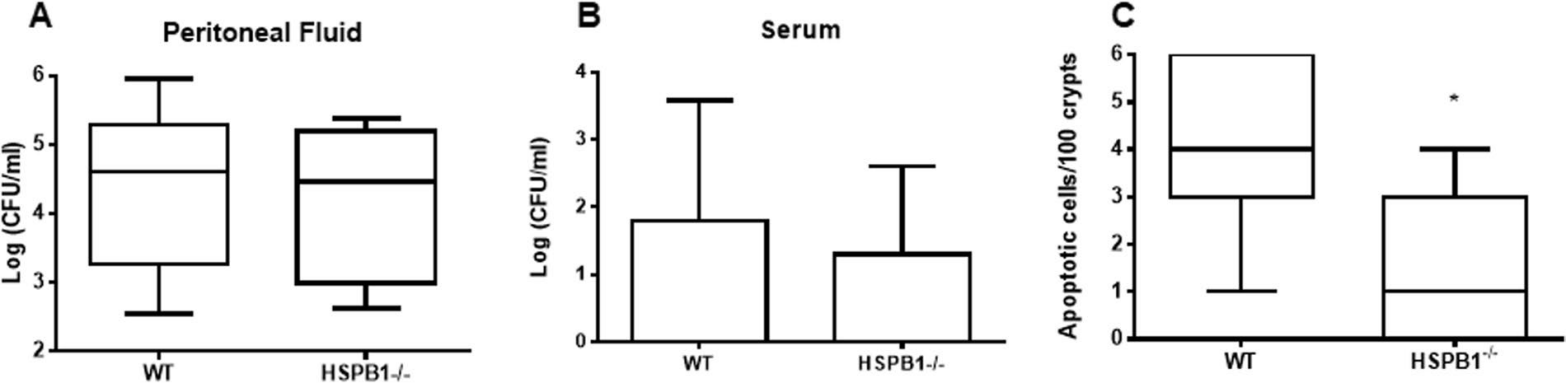
Effect of HSBP1 deletion on bacterial burden and gut apoptosis. Bacterial levels were similar in both the peritoneal fluid (**A**, p = 0.94) and systemic circulation (**B**, (p > 0.99)) in both HSPB1^−/−^ and WT mice (n = 5/group for both). Gut epithelial apoptosis was lower in septic HSPB1^−/−^ mice (**C**, p = 0.02, n = 7/group).

Although bacteria were detected in both the peritoneal cavity and systemic circulation following sepsis, there were no differences in bacterial burden between WT and HSPB1^−/−^ mice (Fig. 8A,B). Intestinal epithelial apoptosis was lower in HSPB1^−/−^ mice than WT mice following CLP (Fig. 8C).

## Discussion

The small 25 kilodalton (kD) HSPB1 protein is a member of the larger HSP family that also includes the well-known and well-characterized HSP70 and HSP90 proteins. As chaperones, these proteins mediate protein-protein interactions that can range in function from cell signaling to protein folding/degradation pathways. The basal and inducible expression of HSPB1 in a wide range of cultured cell models has been extensively studied but the corresponding *in vivo* functional studies have been limited until recently. Despite a wide range of HSPB1 tissue levels from barely detectable to extremely high^5,6^, three independent HSPB1^−/−^ mouse lines have now been generated^8–10^ and no effect of HSPB1 loss on mouse fertility, overall health or longevity has been apparent.

In contrast, the results presented herein demonstrate a crucial role for HSPB1 in the setting of *in vivo* stress. Sepsis is life threatening organ dysfunction caused by a dysregulated host responsive to infection^23^. Sepsis is the third most common cause of death in the United States with at least 270,000 patients dying from sepsis annually^24^. Multiple associative or *in vitro s*tudies indicate that HSPs play an important role in mediating the host response in sepsis^25–27^. HSP70 expression is decreased in the lungs following CLP, and giving parenteral glutamine following CLP improves survival, an effect associated with increased HSP70 expression in multiple tissues^28–30^. This survival benefit is not seen in septic HSP70^−/−^ mice given glutamine^29^. Furthermore, HSP70 induction is impaired in *ex-vivo* lymphocytes of septic patients^31^, and HSP70 is a key determinant of mortality in aged septic hosts^26^. Increased HSP90 is also associated with an acute inflammatory-metabolic stress response in children with sepsis and multiple organ failure^32^, while inhibiting HSP90 ameliorates multiple organ dysfunction syndrome in rats subjected to endotoxemia^33^.

The role of HSPB1 in sepsis is less well understood. Mice overexpressing HSPB1 specifically in cardiac tissue exhibit improved survival following endotoxemia^27^. In addition, riboflavin protects mice from endotoxin-induced shock via expression of HSBP1^34^ and endotoxin-induced endothelial barrier dysfunction correlates with HSPB1 phosphorylation^35^. Importantly, however, endotoxemia does not replicate many of the key components of sepsis^36^ and is therefore not recommended as a clinically-relevant model of sepsis^37,38^. We therefore examined HSPB1^−/−^ mice following CLP to test whether HSPB1 is a critical factor in limiting injury or mortality following sepsis. Strikingly, mortality was nearly twice as high in knockout mice, demonstrating a critical role for HSPB1 that is unmasked only in the setting of severe injury. The mechanisms underlying this alteration in mortality may relate to significant changes in arterial compliance, the adaptive immune system and cytokine production.

Skeletal and aortic smooth muscles both express endogenous levels of HSPB1, the loss of which has no apparent impact on health. However, in skeletal muscle, HSPB1 loss decreases resistance to repetitive muscle fatigue and HSPB1^−/−^ mice display decreased voluntary running^10^. Notably, macroscopic examination of soleus muscle tissue from HSPB1^−/−^ mice failed to reveal any structural alterations and only electron microscopy was able to detect minor myofibril changes^39^. Since *in situ* hybridization studies on HSPB1 expression in mouse tissues identified elevated mRNA levels in the arterial system^6^ and a preliminary microarray experiment from MEFs lacking HSPB1 demonstrated altered elastin levels (data not shown), we examined whether loss of HSPB1 adversely affected arterial structure and function in septic mice. No differences were detected in elastin content and organization and histologic appearance between septic HSPB1^−/−^ and WT mice. In contrast, compliance of carotid arteries in HSPB1^−/−^ mice was significantly increased over physiologic blood pressures compared to carotid arteries from WT mice when measured using biaxial testing *ex vivo* on carotid arteries removed at time of sacrifice. On the surface, this result is surprising since in cardiovascular patients, compliance is thought to be desirable. It is possible, however, that increased compliance may have paradoxically contributed to the higher mortality rates identified in HSPB1^−/−^ mice by having a detrimental impact on vascular tone and blood pressure^40^. Similarly as diastolic pressure predominates the cardiac cycle, small increases in compliance could have profound impacts on the diastolic tone and organ perfusion, particularly when systemically disrupted by the chemokine response as has been found in large animal models of peritonitis^41^. Notably, since the biaxial data demonstrated increased compliance throughout physiologic blood pressures, this suggests that differences persist throughout the cardiac cycle.

Cellular cytokine production is generally considered to be beneficial in sepsis as it allows the host to effectively eradicate pathogen^42^. As such, the decreased production of TNF and IL-2 seen in HSPB1^−/−^ mice represents a potential mechanism for worsened mortality in these animals. It should be noted that despite decreased intracellular cytokine production, there was no difference in bacterial clearance at 24 hours. However, there are numerous studies in which changes specifically in the adaptive immune system improve survival in sepsis, yet this is not associated with alterations in bacterial burden, suggesting a complex role for how perturbations in the adaptive immune system impact mortality^43^. While the increase in IFN γ production in CD4^+^ T cells would be expected to beneficial, the increased mortality seen in HSPB1^−/−^ mice suggests that this mild increase was not sufficient to overcome other detrimental changes in the host response. In addition, the changes identified in HSPB1^−/−^ mice in both T cells and cytokine production are complicated by the observation that HSPB1 is not readily detectable in either human monocytes or T-cells^12^. This suggests a potential indirect effect via still to be determined factors that secondarily impact T cell number and function. Defective inter-cellular signaling from HSPB1 deficient stroma cells or HSPB1 containing exosomes generated from injured cells^44^ are possible factors impacting immune response to HSPB1 but these possibilities have been little explored to date and will require additional tissue-specific gene deletion studies. It should also be noted that there was no difference in bacterial burden detectable in either the blood or peritoneal fluid of WT and HSPB1^−/−^ mice. The concept that the host can protect against infectious disease by reducing the negative impact of infections on host fitness (also known as “disease tolerance”) is a relatively new concept that could potentially help explain the differential mortality in the absence of differential bacterial burden^45^. While the mechanisms underlying the diminished response to infection are complex, damage control mechanisms allow for preservation of functional output of parenchymal tissues, thus maintaining homeostatic parameters within a dynamic range, contributing to survival^46^.

The decrease in gut epithelial apoptosis in septic HSPB1^−/−^ mice compared to WT mice was unexpected. Gut epithelial apoptosis is increased in sepsis^47–49^, and preventing this is associated with improved survival^50,51^. Since *in vitro* HSPB1 studies indicate the protein can block apoptotic pathways^52,53^, we had expected a further increase in gut apoptosis as a possible mechanism for decreased survival in knockout mice. Additionally, knocking out HSP70 alters gut apoptosis in aged septic hosts^26^. Together, this suggests HSP control of apoptosis is complex, with tissue specific effects controlled by still unknown factors.

The mechanism underlying the role of HSPB1 in injury-induced inflammation is not entirely clear. We show in this study that in cultured MEF cells from HSPB1^−/−^ mice, NF-κB signaling initiated by LPS-induced IKb degradation is unperturbed. It does not appear, therefore, that HSPB1 is strictly required for regulating NF-κB activation. Similar results have been reported for IL-1β stimulated HeLa cells in which TAK signaling was reportedly HSPB1 sensitive^12^. The latter report also indicated that mRNA stability of IL-1 stimulated inflammatory mediators COX-2 and IL-6 was substantially altered after HSPB1 depletion. This is consistent with recent discoveries that HSPB1 is required for degradation of the AUF1 protein, which enhances turnover of mRNAs containing ARE elements^54^. Cytokine mRNAs are commonly rich in ARE and AUF1^−/−^ gene mice have increased mouse mortality after endotoxemia due to a hyper-inflammatory response^55^.

The study has a number of limitations. All non-survival experiments were performed 24 hours after sepsis, and temporal changes in parameters examined cannot be known by this experimental design. Specifically, the 24 hour end point may be early for extracellular matrix remodeling to affect arterial mechanics (although they have been reported within 48 hours)^56^. Further, HSPB1^−/−^ mice have life-long genetic alterations that result in subtle findings and potentially other chronic changes that alter the response to acute injury. Next, by choosing to examine common carotid arteries, it is possible that we did not detect an important phenotype in a different major blood vessel, such as the aorta. It is also important to mention that carotid artery compliance was performed *ex vivo* using biaxial testing on arteries removed at time of sacrifice. While this experimental design has the advantage of allowing us to test compliance across an entire spectrum of blood pressures (Fig. 3D), the *ex vivo* nature of this experiment did not allow measurement of compliance *in vivo* at a particular time and physiologic state. Next, mice ranged from 6–18 weeks of age, and even though WT and HSPB1^−/−^ mice were age matched, we cannot rule out an effect of age on our results since older mice would be expected to have larger body weights. Finally, the cause of worsened mortality in septic HSPB1^−/−^ mice cannot be conclusively determined based upon our associative findings.

Despite these limitations, our results indicate that HSPB1 significantly contributes to protection *in vivo* from injury and mortality following CLP. Given the clinical importance of sepsis, the molecular mechanisms and tissue specific functions of HSPB1 require further investigation.

## References

[1] Ikwegbue, P. C. *et al*. Roles of Heat Shock Proteins in Apoptosis, Oxidative Stress, Human Inflammatory Diseases, and Cancer. Pharmaceuticals. (Basel) **11**(1) (2017). 10.3390/ph11010002 PMC5874698 29295496

[2] Yu A (2015). Roles of Hsp70s in Stress Responses of Microorganisms, Plants, and Animals. Biomed. Res. Int..

[3] Zuo D, Subjeck J, Wang XY (2016). Unfolding the Role of Large Heat Shock Proteins: New Insights and Therapeutic Implications. Front Immunol..

[4] Arrigo AP (2017). Mammalian HspB1 (Hsp27) is a molecular sensor linked to the physiology and environment of the cell. Cell Stress. Chaperones..

[5] Klemenz R (1993). Expression of the murine small heat shock proteins hsp 25 and alpha B crystallin in the absence of stress. J. Cell Biol..

[6] Wakayama T, Iseki S (1998). Expression and cellular localization of the mRNA for the 25-kDa heat-shock protein in the mouse. Cell Biol. Int..

[7] Gernold M (1993). Development and tissue-specific distribution of mouse small heat shock protein hsp25. Dev. Genet..

[8] Crowe J (2013). Heat shock protein B1-deficient mice display impaired wound healing. PLoS. ONE..

[9] Huang L (2007). Insights into function and regulation of small heat shock protein 25 (HSPB1) in a mouse model with targeted gene disruption. Genesis..

[10] Huey KA, Hilliard CA, Hunt CR (2013). Effect of HSP25 loss on muscle contractile function and running wheel activity in young and old mice. Front Physiol.

[11] Park KJ, Gaynor RB, Kwak YT (2003). Heat shock protein 27 association with the I kappa B kinase complex regulates tumor necrosis factor alpha-induced NF-kappa B activation. J. Biol. Chem..

[12] Alford KA (2007). Heat shock protein 27 functions in inflammatory gene expression and transforming growth factor-beta-activated kinase-1 (TAK1)-mediated signaling. J. Biol. Chem..

[13] Sinsimer KS (2008). Chaperone Hsp27, a novel subunit of AUF1 protein complexes, functions in AU-rich element-mediated mRNA decay. Mol. Cell Biol..

[14] Ryazantseva NV (2015). Role of heat shock protein 27 in regulation of glutathione system and apoptosis of Jurkat tumor cells and blood lymphocytes. Bull. Exp. Biol. Med..

[15] Baker CC (1983). Evaluation of factors affecting mortality rate after sepsis in a murine cecal ligation and puncture model. Surgery.

[16] Rittirsch D (2009). Immunodesign of experimental sepsis by cecal ligation and puncture. Nat. Protoc..

[17] Rhodes A (2017). Surviving Sepsis Campaign: International Guidelines for Management of Sepsis and Septic Shock: 2016. Crit Care Med..

[18] Gleason RL (2004). A multiaxial computer-controlled organ culture and biomechanical device for mouse carotid arteries. J. Biomech. Eng.

[19] Hansen L (2013). Azidothymidine (AZT) leads to arterial stiffening and intima-media thickening in mice. J. Biomech..

[20] Wang R (2014). Differential mechanical response and microstructural organization between non-human primate femoral and carotid arteries. Biomech. Model. Mechanobiol..

[21] Klingensmith, N. J. *et al*. Epidermal Growth Factor Improves Intestinal Integrity and Survival in Murine Sepsis Following Chronic Alcohol Ingestion. **47**(2), 184 (2017). 10.1097/SHK.0000000000000709 PMC5235949 27465753

[22] Vyas D (2007). Epithelial apoptosis in mechanistically distinct methods of injury in the murine small intestine. Histol. Histopathol..

[23] Singer, M. *et al*. The Third International Consensus Definitions for Sepsis and Septic Shock (Sepsis-3). **315**(8), 801 (2016). 10.1001/jama.2016.0287 PMC4968574 26903338

[24] Rhee, C. *et al*. Incidence and Trends of Sepsis in US Hospitals Using Clinical vs Claims Data, 2009–2014. **318**(13), 1241 (2017). 10.1001/jama.2017.13836 PMC5710396 28903154

[25] Edelman DA (2007). Lipopolysaccharide up-regulates heat shock protein expression in rat lung pericytes. J. Surg. Res..

[26] McConnell KW (2011). The role of heat shock protein 70 in mediating age-dependent mortality in sepsis. J. Immunol..

[27] You, W. *et al*. Cardiac-specific expression of heat shock protein 27 attenuated endotoxin-induced cardiac dysfunction and mortality in mice through a PI3K/Akt-dependent mechanism. **32**(1), 108 (2009). 10.1097/SHK.0b013e318199165d 19106822

[28] Singleton KD (2005). Glutamine attenuates lung injury and improves survival after sepsis: role of enhanced heat shock protein expression. Crit Care Med..

[29] Singleton KD, Wischmeyer PE (2007). Glutamine’s protection against sepsis and lung injury is dependent on heat shock protein 70 expression. Am. J. Physiol Regul. Integr. Comp Physiol.

[30] Weiss, Y. G. *et al*. Cecal ligation and double puncture impairs heat shock protein 70 (HSP-70) expression in the lungs of rats. **13**(1), 19 (2000). 10.1097/00024382-200013010-00004 10638664

[31] Schroeder S (1999). Impaired inducibility of heat shock protein 70 in peripheral blood lymphocytes of patients with severe sepsis. Crit Care Med..

[32] Fitrolaki MD (2016). Increased extracellular heat shock protein 90alpha in severe sepsis and SIRS associated with multiple organ failure and related to acute inflammatory-metabolic stress response in children. Medicine (Baltimore).

[33] Wang YL (2016). 17-DMAG, an HSP90 Inhibitor, Ameliorates Multiple Organ Dysfunction Syndrome via Induction of HSP70 in Endotoxemic Rats. PLoS. ONE..

[34] Shih CK (2010). Riboflavin protects mice against liposaccharide-induced shock through expression of heat shock protein 25. Food Chem. Toxicol..

[35] Hirano S (2004). Endothelial barrier dysfunction caused by LPS correlates with phosphorylation of HSP27 *in vivo*. Cell Biol. Toxicol..

[36] Remick DG (2000). Comparison of the mortality and inflammatory response of two models of sepsis: lipopolysaccharide vs. cecal ligation and puncture.

[37] Fink MP (2014). Animal models of sepsis. Virulence..

[38] Stortz, J. A. *et al*. Murine Models of Sepsis and Trauma: Can We Bridge the Gap? ILAR. J. 1 (2017). 10.1093/ilar/ilx007 PMC5886315 28444204

[39] Kammoun M (2016). The Invalidation of HspB1 Gene in Mouse Alters the Ultrastructural Phenotype of Muscles. PLoS. ONE..

[40] Werner HA, Herbertson MJ, Walley KR (1995). Amrinone increases ventricular contractility and diastolic compliance in endotoxemia. Am. J. Respir. Crit Care Med..

[41] Stahl TJ (1990). Sepsis-induced diastolic dysfunction in chronic canine peritonitis. Am. J. Physiol.

[42] Seder RA, Darrah PA, Roederer M (2008). T-cell quality in memory and protection: implications for vaccine design. Nat. Rev. Immunol..

[43] Chen CW (2017). Cutting Edge: 2B4-Mediated Coinhibition of CD4(+) T Cells Underlies Mortality in Experimental Sepsis. J. Immunol..

[44] Clayton A (2005). Induction of heat shock proteins in B-cell exosomes. J. Cell Sci..

[45] Medzhitov R, Schneider DS, Soares MP (2012). Disease tolerance as a defense strategy. Science.

[46] Weis S (2017). Metabolic Adaptation Establishes Disease Tolerance to Sepsis. Cell.

[47] Fay KT, Ford ML, Coopersmith CM (2017). The intestinal microenvironment in sepsis. Biochim. Biophys. Acta.

[48] Klingensmith NJ, Coopersmith CM (2016). The Gut as the Motor of Multiple Organ Dysfunction in Critical Illness. Crit Care Clin..

[49] Meng M, Klingensmith NJ, Coopersmith CM (2017). New insights into the gut as the driver of critical illness and organ failure. Curr. Opin. Crit Care.

[50] Coopersmith, C. M. *et al*. Overexpression of Bcl-2 in the intestinal epithelium improves survival in septic mice. **30**(1), 195 (2002). 10.1097/00003246-200201000-00028 11902262

[51] Coopersmith, C. M. *et al*. Inhibition of intestinal epithelial apoptosis and survival in a murine model of pneumonia-induced sepsis. **287**(13), 1716 (2002). 10.1001/jama.287.13.1716 11926897

[52] Kennedy D (2017). HSPB1 facilitates ERK-mediated phosphorylation and degradation of BIM to attenuate endoplasmic reticulum stress-induced apoptosis. Cell Death. Dis..

[53] Mo XM (2016). Up-regulation of Hsp27 by ERalpha/Sp1 facilitates proliferation and confers resistance to apoptosis in human papillary thyroid cancer cells. Mol. Cell Endocrinol..

[54] Li ML, Defren J, Brewer G (2013). Hsp27 and F-box protein beta-TrCP promote degradation of mRNA decay factor AUF1. Mol. Cell Biol..

[55] Lu JY, Sadri N, Schneider RJ (2006). Endotoxic shock in AUF1 knockout mice mediated by failure to degrade proinflammatory cytokine mRNAs. Genes Dev..

[56] Gleason RL, Wilson E, Humphrey JD (2007). Biaxial biomechanical adaptations of mouse carotid arteries cultured at altered axial extension. J. Biomech..

